# Safety of outpatient commencement of sotalol

**DOI:** 10.1016/j.hroo.2024.05.003

**Published:** 2024-05-17

**Authors:** Suraya H. Kamsani, Melissa E. Middeldorp, Glenda Chiang, Maria Stefil, Shaun Evans, Mau T. Nguyen, Elnaz Shahmohamadi, Jessica Qingying Zhang, Kurt C. Roberts-Thomson, Mehrdad Emami, Glenn D. Young, Prashanthan Sanders

**Affiliations:** ∗ Centre for Heart Rhythm Disorders, University of Adelaide, Adelaide, South Australia, Australia; † Department of Cardiology, Royal Adelaide Hospital, Adelaide, South Australia, Australia; ‡ National Heart Institute, Kuala Lumpur, Malaysia; § Department of Cardiology, University of Groningen, University Medical Center Groningen, Groningen, The Netherlands

**Keywords:** Sotalol, Drug therapy, Outpatients, Oral loading, QTc, Torsades de pointes

## Abstract

**Background:**

Inpatient monitoring is recommended for sotalol initiation.

**Objective:**

The purpose of this study was to assess the safety of outpatient sotalol commencement.

**Methods:**

This is a multicenter, retrospective, observational study of patients initiated on sotalol in an outpatient setting. Serial electrocardiogram monitoring at day 3, day 7, 1 month, and subsequently as clinically indicated was performed. Corrected QT (QTc) interval and clinical events were evaluated.

**Results:**

Between 2008 and 2023, 880 consecutive patients who were commenced on sotalol were evaluated. Indications were atrial fibrillation/flutter in 87.3% (n = 768), ventricular arrhythmias in 9.9% (n = 87), and other arrhythmias in 2.8% (n = 25). The daily dosage at initiation was 131.0 ± 53.2 mg/d. The QTc interval increased from baseline (431 ± 32 ms) to 444 ± 37 ms (day 3) and 440 ± 33 ms (day 7) after sotalol initiation (*P* < .001). Within the first week, QTc prolongation led to the discontinuation of sotalol in 4 and dose reduction in 1. No ventricular arrhythmia, syncope, or death was observed during the first week. Dose reduction due to asymptomatic bradycardia occurred in 3 and discontinuation due to dyspnea in 3 within the first week. Overall, 1.1% developed QTc prolongation (>500 ms/>25% from baseline); 4 within 3 days, 1 within 1 week, 4 within 60 days, and 1 after >3 years. Discontinuation of sotalol due to other adverse effects occurred in 41 patients within the first month of therapy.

**Conclusion:**

Sotalol initiation in an outpatient setting with protocolized follow-up is safe, with no recorded sotalol-related mortality, ventricular arrhythmias, or syncope. There was a low incidence of significant QTc prolongation necessitating discontinuation within the first month of treatment. Importantly, we observed a small incidence of late QT prolongation, highlighting the need for vigilant outpatient surveillance of individuals on sotalol.

## Graphical abstract

**Figure:**
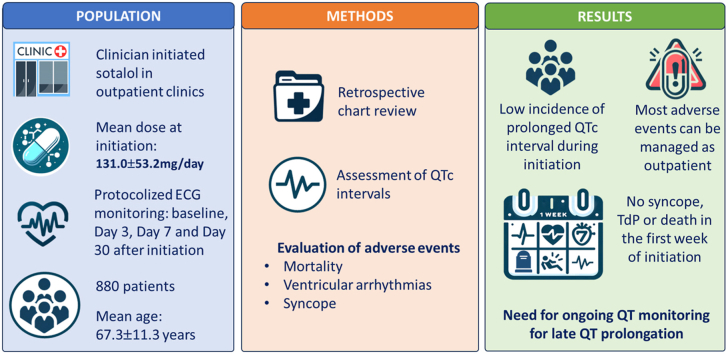

**Key Findings**

▪ Outpatient initiation of sotalol with the dose determined by the clinician in the real-world outpatient clinic setting found a low incidence of corrected QT interval prolongation within the first week of sotalol initiation.
▪ Most patients were safely commenced on the medication, and none experienced syncope, ventricular arrhythmias, or sotalol-related death within the first month of initiation.
▪ Other adverse effects necessitating withdrawal or dose adjustment of sotalol were predominantly managed in the outpatient setting.
▪ A small group of patients developed prolongation of the QT interval late after normal initial monitoring, highlighting the need for ongoing surveillance in those who continue sotalol chronically.

## Introduction

Sotalol is a nonselective β-adrenoceptor antagonist that also has a potent potassium channel–blocking effect. Because of this unique property, it is classified as a class III antiarrhythmic agent in the Vaughan-Williams classification system. Sotalol was first approved by the Food and Drug Administration (FDA) in 1992 and has been mainly used for life-threatening ventricular arrhythmias and maintenance of sinus rhythm in patients with symptomatic atrial fibrillation (AF).^1^, ^2^, ^3^, ^4^, ^5^, ^6^

A significant concern is related to sotalol-induced QT prolongation as a result of delayed repolarization and prolongation of action potential duration mediated by its class III effect.^7^ Sotalol displays a reverse use dependence property, with an inverse relationship between heart rate and QT prolongation, in which QT interval increases as the heart rate reduces.^8^ A torsades de pointes event is thought to occur because of triggered activity caused by early afterdepolarization, and the risk is reported to be between 1% and 4% in patients on sotalol.^9^, ^10^, ^11^, ^12^ Chung et al^13^ found significant arrhythmia complications including new or increased ventricular arrhythmias, bradycardia, and excessive QT prolongation in ∼1 in 5 patients initiated on sotalol during their inpatient surveillance. Proarrhythmic risk has been shown to be higher in patients with renal impairment, left ventricular hypertrophy, and bradycardia and higher in female patients than in male patients.^14^ Previous studies such as the Sotalol, Amiodarone Atrial Fibrillation Efficacy Trial (SAFE-T trial) demonstrated a doubling in mortality for patients prescribed with sotalol compared with placebo, although this was not statistically significant and was observed well beyond the drug initiation period.^15^ A subsequent meta-analysis of 5 randomized controlled trials comprising 1882 participants showed a significant high-certainty evidence of increased all-cause mortality and proarrhythmic effects with sotalol when used for rhythm control in patients with AF.^6^

Because of this proarrhythmic concern, current FDA recommendations outline the need for inpatient monitoring for a minimum of 3 days for the initiation of sotalol.^16^ These requirements result in significant economic implications in using this strategy, with a reported hospitalization cost between US$3278 and US$12,466, mainly because of the cost of the hospital room itself.^17^^,^^18^ More contemporary studies have suggested the safety of outpatient initiation of sotalol therapy in patients with implantable cardiac devices^19^ and no excess mortality in patients treated with sotalol compared with β-blockers.^20^ Here, we aim to determine the safety aspects of sotalol initiation in outpatient settings without continuous rhythm monitoring.

## Methods

### Study design

The electronic medical records of patients treated with sotalol at the Cardiovascular Centre and Advara HeartCare, Adelaide, Australia, during the period from February 2008 to December 2023 were retrospectively examined. The study was reviewed and approved by the Human Research Ethics Committee of the University of Adelaide and conducted in accordance with the guidelines of the Declaration of Helsinki. The study was granted a waiver for informed consent because of the study’s retrospective nature without direct patient involvement or effect on patient care.

### Study population

Patients with the following criteria were included in the study: (1) age >18 years, (2) documented arrhythmias, or (3) sotalol newly initiated in the prespecified outpatient clinics. Patients were excluded if (1) they did not complete at least 1 month of review in the prespecified outpatient clinic, (2) sotalol was prescribed as an inpatient, (3) sotalol was commenced at another center, or (4) if sotalol was used on an as-required basis only.

### Sotalol initiation protocol

The standardized sotalol prescription protocol for our outpatient clinics is as follows: upon receiving the prescription, patients would present to the clinic for an electrocardiogram (ECG) at baseline, day 3, day 7, and 1 month after initiation to determine ECG changes.

### Study protocol

Individual sociodemographic information, anthropomorphic measurements, comorbidities, arrhythmia histories, concomitant medications, and baseline ECG and echocardiogram data were extracted from the electronic medical record. Follow-up ECGs at day 3, day 7, 1 month, and subsequently as clinically indicated were scrutinized, and corrected QT (QTc) intervals were recorded. The QTc interval was calculated from the available 12-lead ECG using the Bazett formula by 3 assessors. *Excessive QTc prolongation* was defined as QTc interval > 500 ms or an increase of >25% from baseline. In the presence of ventricular pacing or bundle branch block (BBB), the modified QTc formula was used on the basis of QT_modified_ = QT_BBB_ − 50% of QRS_BBB_.^21^

### Study end points

The primary outcomes examined were (1) excessive QTc prolongation (>500 ms or >25% increase from baseline) at 3 days, 1 week, and 1 month from sotalol initiation and (2) significant ventricular arrhythmias, syncope, or sotalol-related death in the first month after the initiation of sotalol.

The secondary end points were (1) excessive QTc prolongation at any time point during patients’ follow-up and (2) dose adjustment or cessation of sotalol due to any adverse events within the first month of treatment.

Adverse effects, including the incidence of significant ventricular or brady-arrhythmias, syncope, or death within the first month of sotalol therapy were determined from chart review. *Significant ventricular arrhythmic events* were defined as new or increased premature ventricular complexes or ventricular tachycardia including torsades de pointes, whereas *significant bradyarrhythmia* was defined as a documented heart rate of ≤40 beats/min or pause ≥ 3 seconds, regardless of the presence of symptoms, leading to dose adjustment and/or pacemaker implantation.

### Statistical analysis

Statistical analysis was performed with IBM SPSS Statistics version 28.0 (IBM Corporation, Armonk, NY). Descriptive statistics for continuous variables are presented as mean ± SD or median (interquartile range [IQR]) according to the distribution. Normality of distribution was determined using the Kolmogorov-Smirnov test. Categorical variables are summarized as number and percentage. Differences between patients with and without sotalol-induced QTc prolongation were explored using 2-sample *t* tests for continuous and χ^2^ tests for categorical variables. Fisher exact tests were used with predictors that resulted in low cell counts. A *P* value of <.05 was considered statistically significant.

## Results

### Study cohort and baseline characteristics

**Figure 1:**
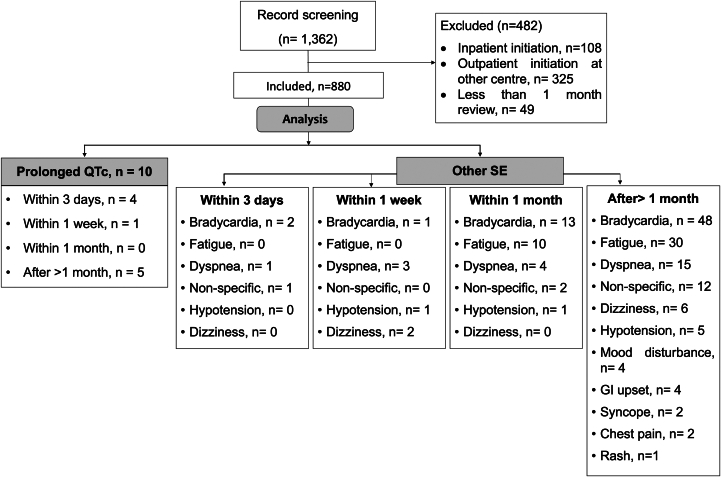
CONSORT diagram of the present retrospective study. GI = gastrointestional; QTc = corrected QT; SE = side effects.

During the study period, 1362 patients received sotalol prescriptions from these centers, with 482 patients excluded from the final analysis because of the following reasons: initiation outside the clinic (inpatient: n = 108; outpatient: n = 325) and did not complete at least 1 month of review in the prespecified outpatient clinic (n = 49). A total of 880 consecutive patients were then included in the study (Figure 1). Their detailed baseline characteristics are outlined in Table 1. The mean age of our study cohort was 67.3 ± 11.3 years, with 523 (59.4%) male patients. The mean daily dosage of sotalol at initiation was 131.0 ± 53.2 mg/d (minimum 40 mg/d, maximum 320 mg/d; median 120 mg/d with IQR 80–160 mg/d). Less than half of the patients (n = 274 [42.5%]) received a initiating daily dosage of ≤120 mg/d, 468 patients (53.2%) received 160 mg/d, and only 138 patients (15.7%) received nonstandard initiating dosage of >160 mg/d. Most patients transitioned to sotalol from other rate control therapies such as β-blockers or calcium channel blockers. The indications for sotalol therapy were AF and atrial flutter in 87.3% (n = 768), ventricular arrhythmias in 9.9% (n = 87), and other supraventricular arrhythmias in 2.8% (n = 25). The rhythm at the initiation of sotalol was sinus rhythm in 61.7% (n = 543), AF in 29.8% (n = 262), atrial flutter/tachycardia in 5.3% (n = 47), and paced rhythm in 3.2% (n = 28) of patients.

**Table 1:** Baseline characteristics of the study population

Characteristic	Total (N = 880)	With QTc prolongation (n = 10)	Without QTc prolongation (n = 870)	*P*
Demographic characteristic				
Age (y)	67.3 ± 11.3	69.7 ± 7.7	67.3 ± 11.3	.51
Male	523 (59.4)	6 (60.0)	517 (59.4)	>.99
Body mass index (kg/m^2^)	29.6 ± 5.9	31.6 ± 6.5	29.6 ± 5.9	.36
Smoking history	209 (23.8)	1 (10.0)	208 (23.9)	.47
Alcohol consumption	250 (28.4)	3 (30.0)	247 (28.6)	>.99
Comorbidities				
Hypertension	469 (53.3)	7 (70.0)	462 (53.1)	.35
Coronary artery disease	221 (25.1)	1 (10.0)	220 (25.3)	.47
Heart failure	163 (18.5)	4 (40.0)	159 (18.3)	.10
Chronic renal impairment	132 (15.0)	2 (20.0)	130 (14.9)	.65
Diabetes mellitus	127 (14.4)	2 (20.0)	125 (14.4)	.64
Asthma/COAD	85 (97)	1 (10.0)	84 (9.7)	>.99
Previous stroke/TIAs	59 (6.7)	2 (20.0)	57 (6.6)	.14
Baseline LVEF (%)	57.4 ± 10.8	52.4 ± 18.4	57.6 ± 10.4	.06
Baseline rhythm				
Sinus rhythm	500 (56.8)	6 (60.0)	494 (56.8)	>.99
Atrial fibrillation	242 (27.5)	3 (30.0)	239 (27.5)	>.99
Paced rhythm	27 (3.1)	1 (10.0)	26 (3.0)	.27
Atrial flutter	39 (4.4)	0 (0)	39 (4.5)	>.99
Atrial tachycardia	4 (0.5)	0 (0)	4 (0.5)	>.99
Baseline heart rate (beats/min)	77.0 ± 23.5	85.3 ± 40.4	76.9 ± 23.2	.26
Baseline QTc interval (ms)	431 ± 32	426 ± 24	431 ± 32	.57
Arrhythmia diagnosis				
Atrial fibrillation/flutter	768 (87.3)	9 (90.0)	759 (87.2)	>.99
Ventricular arrhythmias	87 (9.9)	1 (10.0)	86 (9.9)	>.99
Other supraventricular arrhythmias	25 (2.8)	0 (0)	25 (2.9)	>.99
Previous implantable devices				
Permanent pacemakers	71 (8.1)	1 (10.0)	70 (8.0)	.57
Defibrillator	72 (8.2)	2 (20.0)	70 (8.0)	.19
Loop recorders	16 (1.8)	0 (0)	16 (1.8)	>.99
Baseline eGFR (mL/(min·1.73 m^2^))	74.0 ± 15.6	72.4 ± 20.1	74.0 ± 15.6	.80
Previously failed AAD	150 (17.0)	1 (10.0)	149 (17.1)	>.99
Sotalol daily dosage at initiation (mg/d)	131.0 ± 53.2	168.0 ± 59.0	130.5 ± 53.0	.03

Values are presented as mean ± SD or n (%).

AAD = antiarrhythmic drug; COAD = chronic obstructive airways disease; eGFR = estimated glomerular filtration rate; LVEF = left ventricular ejection fraction; QTc = corrected QT; TIA = transient ischemic attack.

One hundred fifty-nine patients (18.1%) had cardiac implantable electrical devices (CIEDs) before sotalol initiation, with 72 having defibrillators (8.2%), 71 pacemakers (8.1%), and 16 loop recorders (1.8%). Notably, 150 patients (17.0%) had previously failed other class I or III antiarrhythmic drug (AAD) therapies.

### Concomitant medication history

A total of 396 of 880 patients (45.0%) were on concomitant medications that could potentially exacerbate QTc prolongation, with proton pump inhibitors and diuretics being the most frequently prescribed. Other rate-controlling drugs were concomitantly prescribed in 101 patients (11.5%), including digoxin (n = 53), β-blockers (n = 39), and calcium channel blockers (n = 9) (Table 2).

**Table 2:** Concomitant medications during sotalol therapy

Drug class	Medications	No. of patients
QTc-prolonging medications		
Antidepressant	Amitriptyline	17
	Citalopram	22
	Escitalopram	16
	Mirtazapine	10
	Paroxetine	11
	Sertraline	22
	Venlafaxine	8
Antipsychotic	Periciazine	1
	Quetiapine	1
Antirheumatic	Hydroxychloroquine	9
Diuretic	Furosemide	71
	Hydrochlorothiazide	88
	Indapamide	7
Proton pump inhibitor	Esomeprazole	79
	Omeprazole	96
	Pantoprazole	106
	Rabeprazole	19
Miscellaneous	Duromine	1
	Tramadol	3
Rate-controlling medications		
β-Blockers	Atenolol	2
	Bisoprolol	13
	Carvedilol	8
	Metoprolol	14
Calcium-channel blocker	Diltiazem	5
	Verapamil	3
Cardiac glycosides	Digoxin	43

QTc = corrected QT.

### Primary end points: Sotalol-induced QTc prolongation, syncope, ventricular arrhythmia, and 30-day mortality

**Figure 2:**
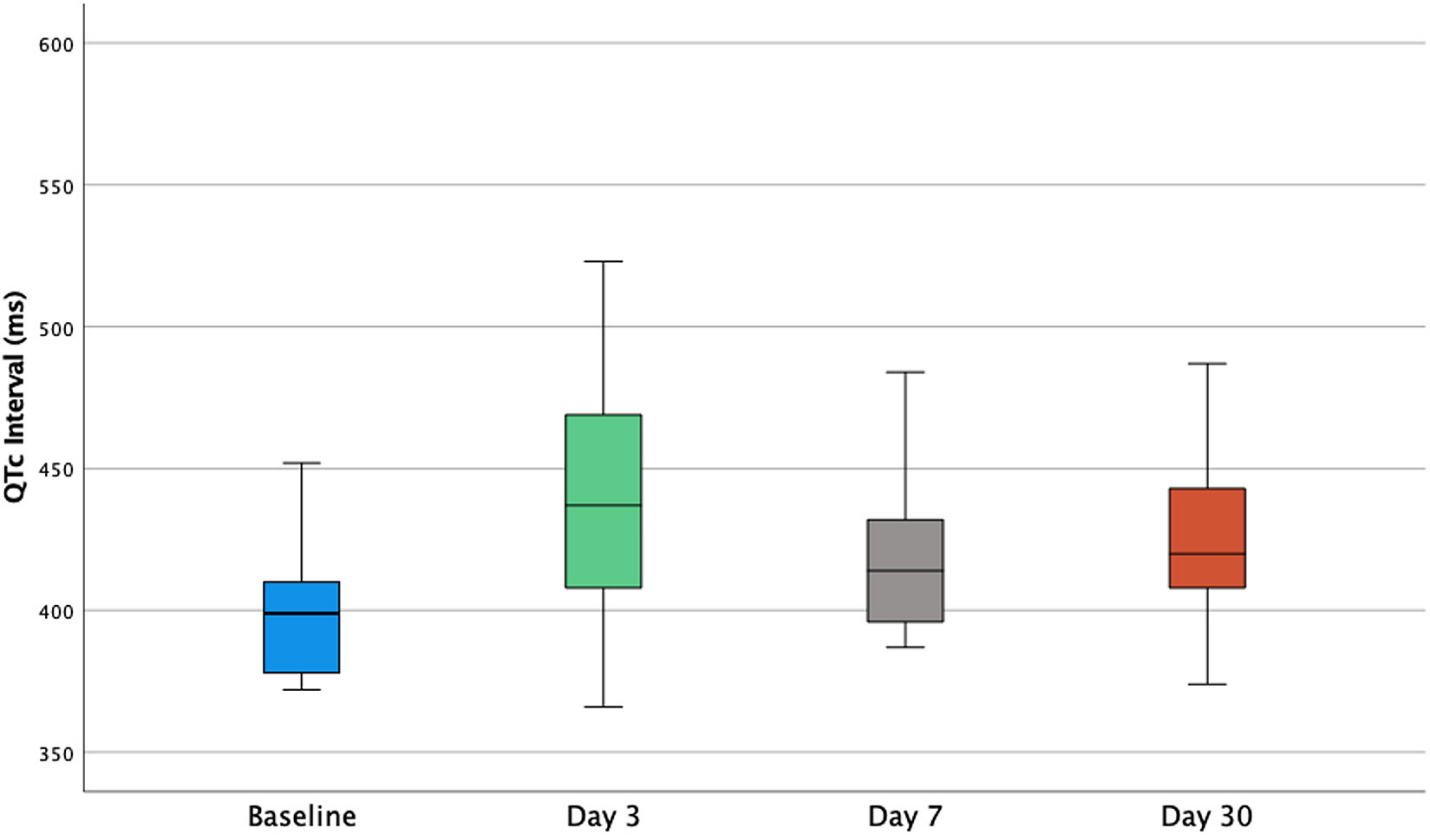
Corrected QT (QTc) intervals at baseline and during sotalol treatment.

The mean baseline QTc interval was 431 ± 32 ms with an increase to 444 ± 37 ms on day 3, 440 ± 33 ms by day 7, and 441 ± 32 ms at 1 month after sotalol initiation (Figure 2). Four patients (0.5%) had to cease their sotalol treatment within the first week because of QT prolongation with QTc interval increasing from 435 ± 14 to 479 ± 15 ms (range 461–493 ms; *P* = .005) (Table 3). Only one of these patients was admitted in the first week of sotalol commencement; initially for observation due to symptomatic AF, QT prolongation was recognized after reversion to sinus rhythm. She was discharged after sotalol cessation without needing further intervention. A further patient had their sotalol dosage reduced from 80 to 40 mg twice daily because of QTc prolongation at day 3 from 453 to 513 ms. The subsequent QTc interval for the patient returned to baseline by day 7, and sotalol was continued at the reduced dose without significant adverse effects. One patient died 12 days after the initiation of sotalol at a dose of 80 mg twice daily. This patient had a background of coronary artery disease and presented with acute myocardial infarction with death due to heart failure. Her baseline QTc interval was 431 ms, and the QTc interval at the time of chest pain presentation was 473 ms. Her death was concluded to be from acute coronary event rather than sotalol related. No other patients experienced syncope, ventricular arrhythmias, or death during the first month of therapy.

**Table 3:** Characteristics of patients with a prolonged QTc interval within the first week of sotalol initiation

Patient no.	Age (y)	Sex	Indication	Dosage at initiation (mg/d)	Concomitant medications	Previously failed class I/III AAD	Presence of a CIED	Baseline eGFR (mL/min)	Baseline LVEF (%)	Baseline HR (beats/min) and rhythm	Baseline QTc interval (ms)	HR (beats/min) and rhythm at the end point	QTc interval at the end point (ms)	Time to the end point (d)	Remarks
1	73	F	PAF	160	Apixaban, olmesartan/HCTZ, PTU	–	–	88	67	68, SR	450	58, SR	490	2	Ceased
2	53	M	NSVT, persistent AF	320	Warfarin, perindopril, atorvastatin	–	ICD	90	40	58, SR	441	59, SR	493	2	Ceased
3	65	M	Persistent AF	160	Aspirin, clopidogrel, warfarin, irbesartan, atorvastatin	–	–	73	62	60, SR	428	57, AF	471	3	Ceased
4	71	M	Persistent AF	160	Warfarin, perindopril, furosemide, pantoprazole	–	ICD	N/A	22	70, SR	489	44, sinus bradycardia	513	3	Dose reduction from 80 to 40 mg twice daily
5	78	M	PAF	160	Rivaroxaban	–	–	N/A	62	54, SR	419	53, SR	461	7	Ceased

AAD = anti-arrhythmic drug; CIED = cardiac implantable electrical device; F = female; GFR = glomerumar filtration rate; HCTZ = hydrochlorthiazide; HR = heart rate; ICD = implantable cardioverter-defibrillator; LVEF = left ventricular ejection fraction; M = male; NSVT = non-sustained ventricular tachycardia; PAF = paroxysmal atrial fibrillation; PTU = propylthiouracil; SR = sinus rhythm.

### Secondary end points: Late prolongation of the QTc interval

Interestingly after extended sotalol therapy, a further 5 patients developed QTc prolongation. Three patients were asymptomatic and QT prolongation was detected on routine clinic ECG between day 36 and day 40 after initiation, of whom 2 developed QT prolongation after the sotalol dose was increased from 80 to 160 mg twice daily. One patient (Table 4, patient 4) was admitted with Takotsubo cardiomyopathy and was detected to have a prolonged QTc interval during hospital admission 60 days after sotalol initiation. In 1 patient (Table 4, patient 5), QT prolongation was detected 1423 days after sotalol initiation. This patient had a pacemaker implanted 1 year into sotalol therapy for sinus node disease and had episodes of nonsustained ventricular tachycardia detected from her device that led to evaluation. Apart from sotalol cessation, the patient underwent further workup with a coronary angiogram, which did not reveal any obstructive coronary artery disease. The detailed descriptions of these late QT prolongation are outlined in Table 4.

**Table 4:** Characteristics of patients with late prolongation of the QTc interval after sotalol initiation

Patient no.	Age (y)	Sex	Indication	Dosage at initiation (mg/d)	Concomitant medications	Previously failed class I/III AAD	CIED	Baseline eGFR (mL/min)	Baseline LVEF (%)	Baseline HR (beats/min) and rhythm	Baseline QTc interval (ms)	HR (beats/min) and rhythm at the end point	QTc interval at the end point (ms)	Time to the end point (d)	Reason for presentation	Remark (s)
1	78	F	Persistent AF	160	Candesartan/HCTZ, esomeprazole, rivaroxaban, spironolactone, ezetimibe, atorvastatin, levothyroxine, salbutamol, budesonide/formoterol	–	–	39	55	66, SR	412	99, SR	510	36	Routine clinic review	Ceased
2	69	M	Persistent AF	160	Apixaban	–	–	N/A	33	148, AF	378	90, AF	471	37	Routine clinic review	Dose reduction from 160 to 80 mg twice daily
3	74	M	Persistent AF	160	Apixaban	–	–	51	41	96, AF	449	58, SR	490	40	Routine clinic review	Ceased
4	69	F	PAF	80	Digoxin, candesartan, dabigatran	–	–	77	55	169, AF	403	88, SR	495	60	Admission with Takotsubo cardiomyopathy	Ceased
5	62	F	PAF	160	Warfarin, thyroxine, esomeprazole, candesartan	Flecainide – rash, amiodarone – hypothyroidism	PPM	89	73	65, A paced	423	75, A paced	453	1423	PPM alert	NSVT detected on PPM, ceased

AAD = anti-arrhythmic drug; CIED = cardiac implantable electrical device; F = female; GFR = glomerumar filtration rate; HCTZ = hydrochlorthiazide; HR = heart rate; LVEF = left ventricular ejection fraction; M = male; NSVT = non-sustained ventricular tachycardia; PAF = paroxysmal atrial fibrillation; PPM = permanent pacemaker; SR = sinus rhythm.

### Comparison between patients with and without sotalol-induced QTc prolongation

Patients with sotalol-induced QTc prolongation were not significantly older than those without (69.7 ± 7.7 years vs 67.3 ± 11.3 years; *P* = .51). There were a similar proportion of male patients in both groups (60% vs 59.4%; *P* > .99). Most of the patients who developed QTc prolongation with sotalol were prescribed the medication for AF (90.0%). Those without QTc change were initiated on sotalol for AF in 87.2% and ventricular arrhythmia in 9.9%. A total of 149 (17.1%) without QTc prolongation had failed other AADs compared with only 1 in the prolonged QTc group. Baseline QTc intervals and renal function were similar between the groups. Of note, patients with QTc prolongation received a higher daily dose of sotalol than did those without (168.0 ± 59.0 mg/d vs 130.5 ± 53.0 mg/d; *P* = .03) (Table 1).

### Secondary end points: Other sotalol-related adverse effects

A total of 41 patients (4.7%) discontinued their sotalol treatment or had dose adjustment due to various adverse effects within the first month of therapy (median days 14; IQR 7–21). Reasons for medication cessation are detailed in Table 5, with the most common symptoms being bradycardia (n = 16) and fatigue (n = 11). Of the 16 patients who were bradycardic while on sotalol, 9 were asymptomatic and had dose reduction without further intervention and 7 had sotalol cessation, with only 2 patients needing pacemaker implantation. Importantly, there were no syncopal events.

**Table 5:** Adverse effects of sotalol therapy within the first month of initiation (n = 41)

Adverse effects	No. of patients	Median days to adverse effects
Bradycardia	16	16.5
Fatigue	11	21
Dyspnea	8	7.5
Nonspecific	3	13
Hypotension	2	12
Dizziness	1	5

## Discussion

Our study cohort of a real-world outpatient clinic setting found a low incidence of QTc prolongation (0.6%) within the first week of sotalol initiation. Most patients (94.8%) were safely commenced on the medication, and none of them experienced syncope, ventricular arrhythmias, or sotalol-related death within the first month of initiation. In addition, other adverse effects necessitating withdrawal or dose adjustment of sotalol were predominantly managed in the outpatient setting. Interestingly, we did have a small group that developed prolongation of the QT interval late after normal initial monitoring, highlighting the need for ongoing surveillance in those who continue sotalol chronically.

Sotalol dose at commencement in the participating clinics was at the physicians’ discretion. Several clinical factors are likely to have been considered and contributed to determining the commencing dose. These include baseline QT interval, prior bradycardia, tolerance to other rate-lowering medications, underlying conduction system disease, left ventricular impairment, renal function, age, and frailty. Similarly, although the clinics have a prespecified ECG monitoring postinititiation, the interpretation and management of the patient was determined by clinician discretion.

Risk factors that could contribute to QT prolongation, including declining renal function, electrolyte imbalance, introduction of concomitant QT-prolonging therapy, uptitration of sotalol, and development of heart failure, should be carefully monitored.^22^ None of the patients in our cohort with late prolongation of the QT interval were initiated on any new therapy within the time period. Renal function and electrolyte levels were not available for review but would have provided some insight into this phenomenon. Two of these patients were able to tolerate lower doses of sotalol and developed QT prolongation only after dose increment. This underscores the importance of QT monitoring not only at initiation but also after each dose escalation.

Historically, sotalol initiation was reported to be associated with significant proarrhythmic risks requiring early intervention.^13^^,^^23^ However, these observations were limited by their small sample sizes and were soon after the introduction of sotalol. In the study by Chung et al,^13^ patients received a much higher initiating dosage of sotalol at 192.0 ± 58.1 mg/d (median dosage 160 mg/d; IQR 80–320 mg/d) as compared with our study, in which the initiating dosage was 131.0 ± 53.2 mg/d. Arrhythmic complications in their study were recorded in 25 patients (20.8%), predominantly attributed to bradycardia (n = 20 [16.7%]), and occurring primarily during overnight monitoring. In contrast, large randomized trials either recruited patients with high-risk profiles such as left ventricular dysfunction or commenced the drug at higher initiating dose than is currently practiced.^24^^,^^25^ A recent Swedish-wide registry study has been particularly encouraging with lower all-cause mortality in patients treated with sotalol than in those with β-blockers after cardioversion for AF.^20^ The present study attempts to give more granular details on the patient profiles and incidence of adverse events during the sotalol initiation period, focusing on the safety aspects of outpatient initiation. Importantly, the findings also reflect changing prescribing behavior that could not be captured in our series with potentially greater caution in those with significant structural heart disease and impaired renal function, together with the more gradual introduction of dosing.

More contemporary studies, primarily in patients with cardiac implantable electronic devices (CIEDs) have proven the feasibility and safety of initiating sotalol in the outpatient setting with certain precautions in place. Most importantly, close monitoring for arrhythmic events is paramount to ensure patient safety. Mascarenhas et al^19^ used the remote monitoring capabilities of CIEDs for safe outpatient initiation of sotalol in their group of patients. The QT interval was assessed from intracardiac electrograms and ECG tracings obtained via manual transmission from the CIEDs at 2-hour post-sotalol dosing, and patients were seen daily for the first 3 days with 12-lead ECGs. Most of their patients (66%) were successfully initiated and maintained on sotalol therapy over a follow-up period of 23 ± 15 months. In our practice, although only 18.2% of patients have prior CIED implantation, all patients were required to obtain a 12-lead ECG on day 3 and day 7 after sotalol initiation, with a resultant 94.8% successful sotalol initiation and maintenance.

The recent introduction of wearable technologies has revolutionized patient care and created the opportunity for intensive virtual monitoring of patients. A few studies have evaluated the use of the FDA-cleared AliveCor KardiaMobile (Mountain View, CA) device to monitor the QTc interval during AAD therapies.^26^, ^27^, ^28^, ^29^ This handheld device has demonstrated accurate QT interpretation in comparison with a conventional 12-lead ECG.^27^^,^^30^ However, these studies enrolled a small number of patients; thus, larger studies are needed to further evaluate the efficacy and reliability of this device in patients receiving AAD therapies or other QT-prolonging medications.

Another important aspect to consider for a safe outpatient sotalol initiation is its dosing and route of administration. Perhaps unsurprisingly, a retrospective study at a large medical center found that a higher initial dosage was associated with a higher incidence of QTc prolongation and more frequent therapy modification.^31^ This aligned with our findings of higher initiating dose in patients with subsequent significant QTc prolongation. However, most of our patients (95.7%) received the standard recommended dose of ≤80 mg twice daily, resulting in an overall lower incidence of adverse events during the initiation period. Indeed, patients who received higher than standard dose were mostly transitioned from other β-blockers. Our study emphasizes the “start low and go slow” approach: sotalol can be safely initiated in a carefully selected group at a low initiating dose and subsequently uptitrated if the ECG remains normal without a prolonged QTc interval after 3 days.

Another multicenter study by Biswas et al^32^ reported that the absence of dose adjustment is a predictor of successful sotalol initiation with factors such as hypertension, use of calcium channel blockers or other β-blockers, and presence of a pacemaker being predictors of dose adjustment. Only 3 of the patients with a sotalol-induced prolonged QTc interval in our cohort had prior CIED implantation, and none received concomitant β-blockers or calcium channel blockers.

Because of the limited nature of our study, we did not examine the cost-effectiveness of our outpatient initiation strategy. However, based on contemporary reports, the average cost per patient per day was US$3611 ± 1049 with an average total cost per 3-day admission of US$12,466 ± 12,652 (median US$8569).^17^ Therefore, an alternative approach with outpatient intravenous (IV) sotalol loading has been considered.^17^^,^^33^^,^^34^ The recently published Feasibility and Safety of Intravenous Sotalol Administered as a Loading Dose to Initiate Oral Sotalol Therapy in Adult Patients With Atrial Fibrillation (DASH-AF) trial found that rapid IV sotalol loading for the treatment of AF is safe and effective without a significant increase in adverse events.^34^ They also reported a potential cost savings of up to US$3500.68 per admission by using this approach as compared to a 3-day inpatient observation with oral sotalol. Another multicenter observational study (the Prospective Evaluation Analysis and Kinetics of IV Sotalol [PEAKS] Registry) supported the safety of IV sotalol loading with only 2 premature termination in their cohort of 167 patients because of bradycardia and hypotension.^35^ The rate of QT prolongation reported was low (2.4%), and 94% of patients were successfully discharged on the target oral sotalol dose with a mean hospital stay of only 1.1 days. IV sotalol is, however, more expensive, costing US$2734 compared to US$27 for a 3-day oral loading. This approach also involved the use of supervised telemetry beds for at least 6 hours as opposed to a total outpatient-based strategy as demonstrated in this study. Future research in the cost analysis of oral sotalol initiation as an outpatient with the use of conventional ECG monitoring, mobile cardiac outpatient telemetry, or other wearable technology is warranted.

The findings of this study have important clinical and health economic consequences in the context of the current requirements for continuous inpatient monitoring.^16^^,^^36^ The absence of any clinically significant event in the first 3 days in our series strongly argues against the current strategy of continuous monitoring, provided a standardized outpatient protocol of review is followed. Importantly, our series also suggests the need for protocol-driven longer-term outpatient ECG monitoring with a small but real incidence of late QT interval prolongation. Frequent medication review, surveillance of renal function, serum potassium and magnesium levels, as well as assessment of cardiac function are equally important to reduce the risk of QT prolongation during long-term sotalol therapy.

### Study limitations

The present study is limited by its observational and retrospective nature. This study design precludes the use of predetermined initial dose and dose adjustment or cessation criteria. Importantly, it precludes the documentation of exclusions used by the clinician before initiating sotalol. In addition, there were 49 patients who were commenced on sotalol in the participating clinics but their care transferred to other heath networks where we do not have follow-up data. Nevertheless, our data represent a real-world setting, with all patients being cared for by certified electrophysiologists. We also included all consecutive patients initiated on sotalol during the study period. Therefore, this minimizes the possibility of selection or ascertainment bias. It is plausible that the present study does not have adequate power to detect a statistically significant difference between the groups because of the very low rate of QTc prolongation in our cohort. Nonetheless, the data obtained from this study could be used to design larger prospective trials examining the safety of sotalol initiation in outpatient settings.

## Conclusion

The study findings demonstrated that sotalol could be safely commenced in an outpatient setting with strict monitoring in place without documented sotalol-related death, ventricular arrhythmias, or syncope within the first month of therapy. We also observed a very low rate of QTc prolongation, with most patients managed as outpatients without needing urgent inpatient care. The same observation can be made with other adverse effects, notably bradycardia, negating the benefit of short inpatient monitoring as previously advocated.

## Disclosures

Dr Emami reports that the 10.13039/501100001786University of Adelaide has received on his behalf consulting fees from 10.13039/100004374Medtronic and 10.13039/100007497Biosense Webster. Dr Sanders reports having served on the advisory board of Medtronic, Abbott Medical, Boston Scientific, PaceMate, and CathRx. Dr Sanders reports that the 10.13039/501100001786University of Adelaide has received on his behalf lecture and/or consulting fees from Medtronic, Boston Scientific, and 10.13039/100000046Abbott Medical. Dr Sanders reports that the 10.13039/501100001786University of Adelaide has received on his behalf research funding from Medtronic, Abbott Medical, 10.13039/100008497Boston Scientific, and MicroPort. All other authors have no disclosures.
